# Detailed analysis of therapy-driven clonal evolution of *TP53* mutations in chronic lymphocytic leukemia

**DOI:** 10.1038/leu.2014.297

**Published:** 2014-10-28

**Authors:** J Malcikova, K Stano-Kozubik, B Tichy, B Kantorova, S Pavlova, N Tom, L Radova, J Smardova, F Pardy, M Doubek, Y Brychtova, M Mraz, K Plevova, E Diviskova, A Oltova, J Mayer, S Pospisilova, M Trbusek

**Affiliations:** 1 Center of Molecular Medicine, Central European Institute of Technology, Masaryk University, Brno, Czech Republic; 2 Department of Internal Medicine – Hematology and Oncology, University Hospital Brno and Faculty of Medicine, Masaryk University, Brno, Czech Republic; 3 Department of Pathology, University Hospital Brno and Faculty of Medicine, Masaryk University, Brno, Czech Republic

## Abstract

In chronic lymphocytic leukemia (CLL), the worst prognosis is associated with *TP53* defects with the affected patients being potentially directed to alternative treatment. Therapy administration was shown to drive the selection of new *TP53* mutations in CLL. Using ultra-deep next-generation sequencing (NGS), we performed a detailed analysis of *TP53* mutations' clonal evolution. We retrospectively analyzed samples that were assessed as *TP53*-wild-type (wt) by FASAY from 20 patients with a new *TP53* mutation detected in relapse and 40 patients remaining *TP53*-wt in relapse. Minor *TP53*-mutated subclones were disclosed in 18/20 patients experiencing later mutation selection, while only one minor-clone mutation was observed in those patients remaining *TP53*-wt (*n*=40). We documented that (i) minor *TP53* mutations may be present before therapy and may occur in any relapse; (ii) the majority of *TP53*-mutated minor clones expand to dominant clone under the selective pressure of chemotherapy, while persistence of minor-clone mutations is rare; (iii) multiple minor-clone *TP53* mutations are common and may simultaneously expand. In conclusion, patients with minor-clone *TP53* mutations carry a high risk of mutation selection by therapy. Deep sequencing can shift *TP53* mutation identification to a period before therapy administration, which might be of particular importance for clinical trials.

## Introduction

In chronic lymphocytic leukemia (CLL), patients harboring *TP53* defects represent a major challenge concerning the effective treatment.^1^ *TP53* mutation and/or 17p deletion severely impede response to chemotherapy,^2, 3^ and affected patients also manifest short clinical responses to its combination with rituximab.^4, 5^ Although alemtuzumab is supposed to act independently on p53, the response rates in monotherapy are far from satisfactory in chemorefractory patients.^6^ The inability of mutated p53 protein to induce apoptosis properly seems to be a primary reason for the observed resistance to treatment.^7^ The p53 dysfunction is also the major cause of genomic instability in CLL cells,^8^ which leads to the acquisition of other genomic variants available for further selection.

*TP53* gene defects have been observed as primarily subclonal events in CLL patients, often emerging at later disease stages.^9^ The frequency of *TP53* defects at diagnosis or before first therapy is only between 5 and 15%,^2, 3, 10, 11^ but the proportion of affected patients is significantly higher after treatment and has been reported to be as high as 44% in a fludarabine-refractory cohort.^12^ Clonal evolution of genetic abnormalities including *TP53* defects is well evidenced in CLL. Recent studies have illustrated the development of 17p and 11q deletions during the disease course, and associated clonal evolution of new 17p deletions with the presence of foregoing therapy.^13, 14^ Concerning *TP53* mutations, well-documented cases of their acquirement under the pressure of chemotherapy have also been reported by us and independently by others.^15, 16, 17, 18^ This led to the suggestion that *TP53* mutations should be investigated before each therapy in CLL patients.^19^

Next-generation sequencing (NGS) technologies currently enable mutation analyses in cancer patients with previously unattainable sensitivity, reaching as far as fractions of percentages. The clinical significance of minor-clone *TP53* mutations has recently been demonstrated by Rossi *et al.*^18^ Therefore, we utilized this powerful tool to study the clonal evolution of *TP53* mutations in detail. We used an amplicon ultra-deep NGS approach with a high coverage to reach maximum sensitivity, and we used a highly accurate proof-reading polymerase to minimize the sequencing errors. The aims of this NGS-based study were to disclose (i) whether minor *TP53*-mutated clones had already been present before the preceding therapy, and if yes, (ii) whether some patients, who are *TP53*-wt after therapy, harbor minor *TP53* mutations that are not selected. These two issues should address whether NGS is capable of identifying patients at risk of *TP53* mutation selection by treatment.

## Materials and methods

### Patients' cohort

The study was performed on CLL patients' peripheral blood samples at the University Hospital Brno (with written informed consent provided in accordance with the Declaration of Helsinki). Four common cytogenetic aberrations including 17p deletions were detected by Interphase fluorescent *in situ* hybridization using probes from MetaSystems (Altlussheim, Germany), and were classified according to the hierarchical cytogenetic model.^20^ *TP53* mutations were identified by yeast functional analysis (FASAY) coupled to Sanger sequencing of DNA templates from red colonies bearing non-functional p53.^16^ Patient selection criteria for NGS analysis were: Cohort I: (i) *TP53* mutational status change from wild-type (wt) to mutated documented using FASAY; (ii) only one therapy applied between the last *TP53*-wt examination and new *TP53* mutation detection. This criterion was established to enable the tracking of clonal evolution during just one relapse; (iii) available DNA from the time when the sample was *TP53*-wt. Cohort II: (i) available results of consecutive FASAY analyses performed in relapse(s) with no *TP53* mutational status change; (ii) DNA available from the period preceding therapy (Supplementary Figure 1).

### Statistical analyses

Fisher's exact test was used to assess the association between categorical variables. Mann–Whitney test was used to compare the continuous variables. Wilcoxon signed-rank test was used for paired comparison of mutation numbers. Survival analysis and time to mutation detection were calculated using the Kaplan–Meier survival estimator. Overall survival was assessed from the date of diagnosis; only disease-related death was considered as an event. Time to mutation detection was assessed from the date of diagnosis to the date of new *TP53* mutation detection (event) or the last *TP53*-wt examination (censored).

Median survival, time to mutation detection, differences between the curves, and hazard ratios were evaluated by the log-rank test using the GraphPad Prism version 5.00 for Windows (GraphPad Software, San Diego, CA, USA).

### Ultra-deep NGS

NGS analysis was performed on MiSeq (Illumina, San Diego, CA, USA) using gDNA from cryopreserved peripheral blood separated CD19+ B-lymphocytes or mononuclear cells; the percentage of CLL cells (CD5+CD19+) was assessed using flow cytometry and was >80% in all cases. In all, 25 ng of patient DNA was amplified with highly accurate proof-reading Q5 Polymerase (New England Biolabs, Ipswich, MA, USA) using *TP53* exon-specific primers (Supplementary Table 1). The experimental design and reaction conditions followed the manufacturer recommendations. Briefly, PCR products were pooled, purified with Agencourt AMPure XP (Beckman Coulter, Brea, CA, USA), and quantified using Qubit dsDNA HS Assay Kit (Life Technologies, Waltham, MA, USA). The purified amplicon mixes were diluted to a total amount of 1 ng. The indexed paired-end library was prepared with Nextera XT DNA Sample Preparation Kit (Illumina) and sequenced using MiSeq Reagent Kit v2 300 cycles (Illumina). To avoid cross-contamination, samples obtained from the same patient in different time periods were sequenced in separate runs. Amplicons and libraries for each run were prepared separately. The median coverage per base achieved was 31 599 reads (range 2601–177 021).

An in-house bioinformatics pipeline was established to call the sequencing variants. For read preprocessing and alignment, we used CLC Genomic Workbench (Qiagen, Hilden, Germany). Variant calling was performed using the deepSNV R-package^21^ with a statistical approach applying the shearwater algorithm to compute Bayes classifiers based on a betabinomial model.^22, 23^ By the reproducibility test, we disclosed that we were able to reliably distinguish point mismatches and ⩾2 nt insertions/deletions (indels) at the level of 0.2% of variant reads, and 1-nucleotide deletions at the level of 1% of variant reads as these may be artificially introduced during the sequencing and alignment process. For further details, see Supplementary material. Moreover, to evaluate the established pipeline, 20 control samples (*TP53* exons 4–10) derived from healthy individuals were sequenced and no alteration in any sample was observed on the above-mentioned detection limits.

## Results

### Consecutive *TP53* mutational analysis confirms the prominent impact of newly acquired *TP53* mutations on survival

**Figure 1:**
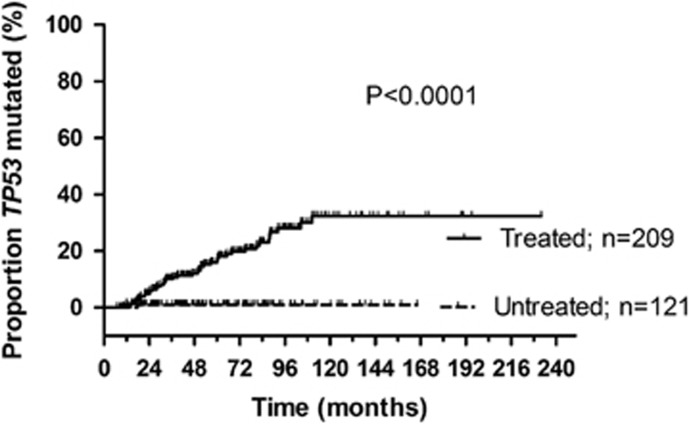
Time from diagnosis to *TP53* mutation acquisition. Patients with *TP53*-wt status at first investigation were repeatedly tested. Time to mutation detection was assessed from the date of diagnosis to the date of new *TP53* mutation detection (event) or the last *TP53*-wt examination (censored). Patients treated during the follow-up acquired new *TP53* mutation significantly more often than untreated patients.

Consecutive *TP53* mutation investigation using FASAY was performed in 330 patients in at least 2 serial samples. All patients harbored intact *TP53* gene at the time of the first analysis (for patients' characteristics, see Supplementary Table 2). Among 121 patients who did not receive any therapy during the follow-up, new *TP53* mutations were observed in only one patient (median follow-up of the group 50 months). In contrast, analyses performed at the time of relapse after one or several therapy lines (*n*=209 patients; median follow-up of the group 61 months) identified new *TP53* mutation(s) in 43 patients. Altogether, the risk of *TP53* mutation acquisition at 5 years after diagnosis was 1% in untreated vs 17% in treated patients (hazard ratio 0.25 (95% CI 0.13–0.47; *P*<0.001)) (Figure 1).

**Figure 2:**
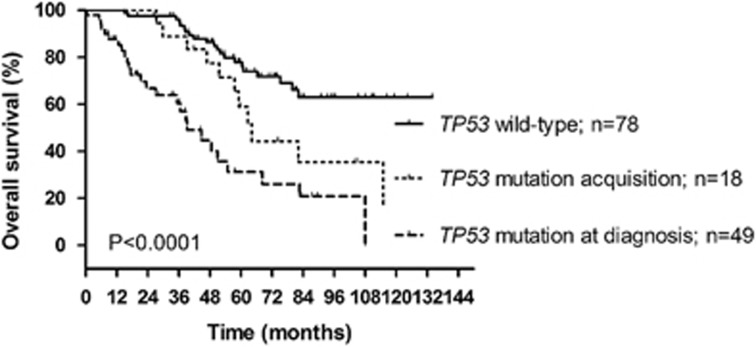
Overall survival according to the *TP53* mutational status in relapse. Overall survival from diagnosis in patients who acquired a new *TP53* mutation at relapse (*n*=18; median survival 64 months) in comparison with patients who remained *TP53*-wt at relapse (*n*=78; median survival undefined; pairwise comparison *P*=0.03), and patients who harbored *TP53* mutation already at diagnosis (*n*=49; median survival 39 months; pairwise comparison *P*=0.02). Only patients with *TP53* status examined at diagnosis or 12 months thereafter were included. All patients included in the analysis underwent treatment and patients having *TP53*-wt status at diagnosis were repeatedly tested for *TP53* mutation presence at subsequent relapse(s).

To assess the importance of *TP53* mutation status change from wt to mutated, we used our cohort analyzed using FASAY and compared the overall survival from diagnosis in patients who acquired a new dominant mutation in relapse with patients who remained *TP53-*wt in relapse, and patients who already harbored *TP53* mutations at diagnosis (Figure 2). The overall survival was significantly reduced in the group of patients who had selected *TP53* mutations compared with patients assessed as wt in relapse (*P*=0.03). The shortest survival was noted for patients with *TP53* mutations already detected at diagnosis.

### NGS analysis reveals the presence of minor mutated clones before their therapy-driven selection

In the first part of our retrospective study, we focused on 20 patients who had acquired a new *TP53* mutation in relapse, as assessed by FASAY coupled to Sanger sequencing (Cohort I; Sample 2). In these patients, we used ultra-deep NGS to examine samples taken before the preceding therapy, which showed *TP53*-wt status using Sanger sequencing and FASAY (Sample 1). In 10 patients, these retrospective samples were treatment naïve (Cohort IA), while the remaining 10 patients had already been pretreated at the time of NGS analysis (Cohort IB) (Table 1). The schematic visualization of samples' inclusion criteria is shown in Supplementary Figure 1. The mutations analyzed consisted of 16 missense mutations, 2 non-sense mutations and 2 deletions and were hence representative of the p53 mutation profile in CLL.^24^ To decipher *TP53* mutagenesis, we sequenced not only the affected regions but also all commonly mutated exons 4–9 (ref. 24) with a high median coverage for the positions containing mutations (25 709 reads; range 5245–64 979). We were able to detect minor-proportion mutations in 18/20 samples (90%), with a proportion of 0.20–3.71% of the reads showing mutations. In 2 of the 18 patients, we surprisingly detected other *TP53* mutations that had not undergone expansion. The results are summarized in Table 1, for details see Supplementary table 3.

Interestingly, in addition to the presumed retrospective mutations, we also identified other minor-proportion *TP53* mutations in both treatment-naïve and pretreated samples (10/20 patients; 2–6 mutations per patient) (Table 1). It indicates that in a proportion of patients, there is a pool of *TP53* mutations available for therapy-driven selection.

We next intended to investigate whether the minor *TP53*-mutated subclones detectable by NGS in pretreated samples and undergoing selection in subsequent relapse (Cohort IB) had already been present before first therapy. Therefore, we used NGS in four available treatment-naïve samples (patients no. 149, 365, 542 and 1043) and confirmed the presence of respective mutation in one of them (patient no. 1043—mutation c.844C>G (p.R282G) detected in 0.2% of NGS reads). This observation suggests that preexisting mutations may expand after the first but also after subsequent therapies at least in some patients.

### Minor *TP53* mutations detectable before therapy are rare in patients remaining *TP53*-wt at relapse

As the next step, we analyzed 40 samples taken before first treatment in patients showing wt-*TP53* status at relapse after one or several therapy lines (Cohort II). These cases were selected from the cohort of relapsing patients, and the inclusion criteria were chosen to collect the cohort with biological and clinical characteristics matching Cohort I (Table 2; Supplementary Figure 1). In this experiment, besides exons 4–9, exon 10 was also sequenced as it may occasionally harbor mutations.^24^

We found *TP53* mutation in only 1 of the 40 patients (2.5%). Specifically, the mutation c.797G4A (p.G266E) was detected in 0.55% (148/32 973) of sequencing reads, and its presence was verified by an independent NGS run. This mutation did not undergo a clonal expansion during the disease course despite several treatment lines—the patient was treated consecutively with three distinct therapy lines (FCR, Alemtuzumab and Rituximab+Dexamethasone) and achieved two complete remissions. In the last available sample from the time of relapse after Rituximab+Dexamethasone treatment (follow-up 47 months) the same mutation was present in 1.4% of reads.

### Clonal selection frequently affects multiple TP53 mutations simultaneously

**Figure 3:**
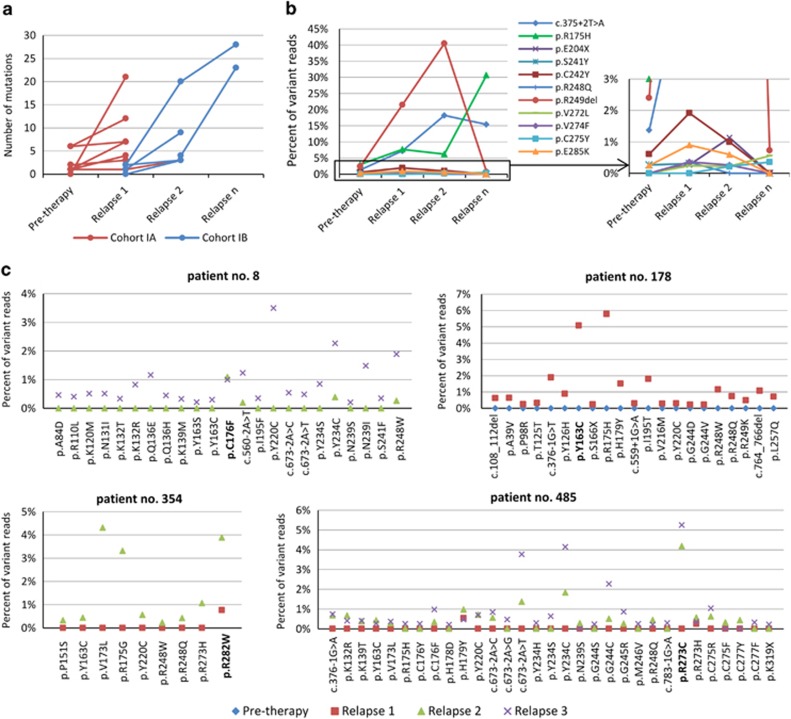
Kinetics of multiple mutations in subsequent samplings. (**a**) Increase in number of mutations detectable using NGS during the disease course. All patients form Cohort I with repeated NGS analysis are shown (*n*=14). (**b**) Dynamics of clonal evolution in patient no. 820. Clone bearing mutation p.249del that was detected at Relapse 2 using FASAY first expanded and was later outgrown by another mutation p.R175H. Splicing mutation c.375+2T>A slightly expanded and coexisted as a minor subclone. Subclonal dynamics of additional minor clones present below 2% is shown in detail. (**c**) Examples of patients with no prominent expansion of one mutation is shown. Proportion of variant reads in individual disease time points is illustrated. Mutation detected using FASAY in the second sampling is highlighted in bold.

As emerged from the previous analyses, multiple minor-clone *TP53* mutations are commonly observed in CLL patients. To further explore this phenomenon in relation to the expansion of major mutations, we performed ultra-deep NGS of *TP53* gene in samples taken at relapse(s) (Sample 2 in Supplementary Figure 1). For this analysis, we had chosen the following patients from Cohort I: (i) six patients with more than one mutation detected in sample 1; (ii) six patients with a single mutation detected in sample 1; and (iii) two patients with no mutation detected in sample 1. Furthermore, the patient with a single non-expanding mutation from Cohort II was also included. An increase in the number of mutations compared with the preceding samples was observed in 13/14 patients from Cohort I (Table 3; Figure 3a). In the paired analysis restricted to samples taken before the first therapy and in the first relapse (Cohort IA), a significant increase in the number of mutations per patient was observed (mean number of mutations per patient 2.1 vs 6.7; *P*=0.02). In the patient from Cohort II, only one mutation was found in both samples.

Regarding the evolution of individual subclones, the most frequently observed event (7/14 patients) was the clear expansion of one mutation from minor to dominant clone accompanied with the occurrence of additional minor *TP53*-mutated clones. In addition to that, we also observed other specific situations: (i) in one case the consecutive selection of two different dominant *TP53* mutations at the first and then the subsequent relapse was noted (one mutation replaced by the other) (patient no. 820; Figure 3b); (ii) in four patients there was not a prominent clonal expansion of one mutation, but multiple clones expanded simultaneously (patients no. 8, 178, 354 and 485; Figure 3c); (iii) one patient underwent only a very slight expansion of a single minor-clone mutation in the first relapse (from 0.2 to 1.46% reads), in the second relapse the proportion of the mutation also increased only slightly (to 2.82% reads) and two other minor-proportion mutations appeared (patient no. 503; Table 3). The results summarizing the rise in the number of mutations in all performed NGS analyses are recapitulated in Supplementary Table 3.

Since the analysis of mutated patients disclosed an increased occurrence of minor *TP53* mutations after treatment, we further analyzed 15 randomly selected patients from Cohort II after 1–4 therapy lines using NGS. No *TP53* mutations were observed in any patient.

### Molecular features of mutations

In total, we identified 148 mutations in 21 patients (Supplementary Table 3) in all the NGS analyses performed. The mutation profile is shown in Supplementary Figures 2 and 3. Compared with the reference study on *TP53* mutation profile in CLL^24^ our results showed the following: (i) a similar proportion of missense mutations (79 vs 74% *P*=0.4) and non-sense mutations (both studies 4%); (ii) the same frequency of mutations at major hot spot codons (175, 179, 220, 248, 273 and 281) (20% of all mutations in both studies); (iii) a significantly higher proportion of splice-site mutations (9 vs 2% *P*=0.005) and, on the other hand (iv) a significantly lower frequency of indel mutations (7 vs 20% *P*=0.0003). Concerning point mutations, transitions represented 61% with only 29% of them (17% of all mutations) occurring at CpG sites. The G-A transitions at CpG predominated C-T transitions (G-A:C-T ratio 2:1). The lower proportion of CpG transitions and the prevalence of G-A exchanges coincided with the reference study.^24^

**Figure 4:**
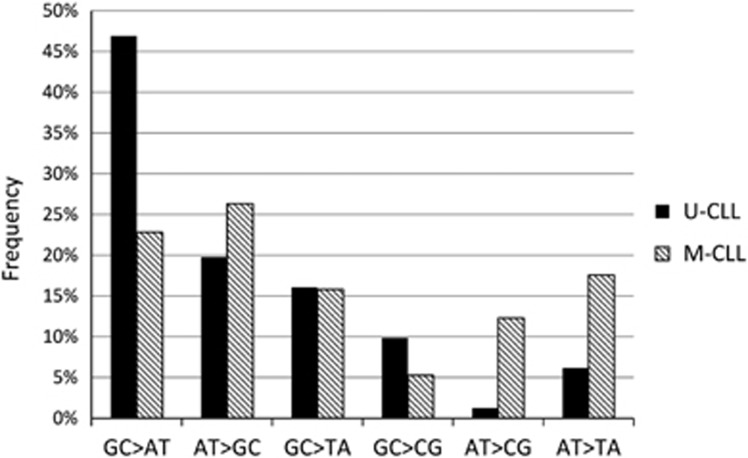
Comparison of *TP53* mutation profile in patients with unmutated *IGHV* (U-CLL) vs mutated *IGHV* (M-CLL). Percentage from all point mutations shown.

Comparison of *TP53* mutation profiles in cases with unmutated immunoglobulin heavy chain gene (*IGHV*; U-CLL) vs mutated *IGHV* (M-CLL) showed no difference in mutation frequency within sequence motif (RGYW/WRCY) recognized by activation-induced cytidin deaminase in U-CLL vs M-CLL (20 vs 18% of point mutations *P*=0.8). In M-CLL, a significant prevalence of alterations in A:T pairs was found compared with U-CLL (56 vs 27% of point mutations; *P*=0.0008). The A:T alteration predominance was the most prominent in case of A:T>C:G transversions (12% in M-CLL vs 1% in U-CLL; *P*=0.009; Figure 4).

As the number of mutations increased after therapy, we also compared the molecular profile of mutations detected in pretherapy samples only (*n*=24) with mutations that occurred exclusively after treatment (*n*=103) and we did not observe any significant differences regarding the proportion of hot spot mutations, transversion-to-transition ratio, proportion of transitions at CpG sites and G:C to A:T ratio (data not shown).

## Discussion

The mechanisms leading to p53 mutation acquisition and accumulation in CLL are poorly understood. The direct induction of *TP53* mutations by DNA-damaging chemotherapy, namely alkylating agents, has been suggested.^25^ In contrast, a large collaborative study involving 268 p53 mutations indirectly showed that the impact of therapy on *de novo* mutation induction is unlikely, as mutation spectra are similar in untreated and treated patients.^24^ This observation, however, may not serve as definitive proof of the neutral impact of therapy on *TP53* mutagenesis, since similar mutations could evolve through different mechanisms. The current progress in highly sensitive techniques, specifically in ultra-deep NGS, allows the possibility to explore whether therapy merely selects *TP53* mutations present in minor CLL clones before drug administration. Moreover, identifying *TP53* defects as early as possible during their evolution may represent a significant achievement in the clinical management of high-risk CLL, since *TP53*-deffective patients could be offered alternative treatment.^1^ The clinical impact of minor-proportion *TP53* defects is currently a matter of debate.^18, 26, 27^ Their relevance for relapse development is supported by the actual number of mutated cells. For instance, at common pretherapy leukocytosis achieving 100 × 10e9 per liter with 90% CLL cells, a patient harbors approximately 4.5 × 10e11 CLL cells in peripheral blood, not considering other organs like the spleen. In this case, a 1% *TP53* mutation corresponds to ~4.5 × 10e9 cells. Moreover, the clinical significance of small *TP53*-mutated clones under the detection limit of Sanger sequencing have very recently been manifested by the study of Rossi *et al.*^18^ showing their similar unfavorable prognostic impact compared with clonal *TP53* defects.

With this report, we focused on two principal issues: (i) exploration of *TP53*-mutated clone evolution and (ii) assessment of NGS utilization in *TP53* mutation expansion prediction in clinical practice. Both these issues are important with respect to the clear negative impact of newly acquired *TP53* mutations on patients' prognosis, which was evidenced by Rossi *et al.*^28^ using time-dependent Cox regression analysis, and is also confirmed here by survival analysis of patients with new mutations.

Concerning the clonal evolution, we documented that the risk of new *TP53* mutation acquisition at 5 years after diagnosis is 17% in patients requiring treatment, contrasting with 1% in untreated patients (*P*<0.001), and we confirmed that selection of preexisting mutated clones by therapy is the predominant mechanism for *TP53* mutations' accumulation. Moreover, we showed that mutations expanding during relapse are detectable before the preceding therapy in the majority of patients. Admittedly, based on our study we cannot entirely exclude that at least some *TP53* mutations are the consequence of DNA damaging drugs^25^ since many minor-proportion mutations were undetectable before first therapy despite using ultra-deep NGS. Although we have not observed any profound difference in the mutation profile of these mutations compared with the mutations present before treatment, they could be induced by therapeutic agents or spontaneous mutagenesis during relapse. Alternatively, they may be present in a very low proportion of leukemic cells under the NGS detection limit.

Our study independently confirms two recent reports^18, 29^ showing that in a proportion of patients there are multiple minor-clone *TP53* mutations (under the Sanger sequencing detection limit). These mutations may or may not accompany a major clonal mutation.^29^ We noted the presence of multiple *TP53* mutations in patients with clonal selection of dominant *TP53* mutations, and also in patients with dominant *TP53* mutation detected at diagnosis (7/10 patients; data not shown). We further observed that selection may affect not only single *TP53*-mutated minor clone, but also in some patients multiple mutations simultaneously. In fact, at least some cases without prominent expansion of one mutation underwent a slight selection of a burden of different *TP53*-mutated clones that are not detectable by Sanger sequencing. Using FASAY, these patients were assessed as ‘mutation acquisition' since with this methodology the overall percentage of red colonies equals the sum of all mutations present.

The striking aspect of our study is the actual number of multiple *TP53* mutations, as according to our observation even tens of mutations may be present in individual patients. Our conclusion that these multiple alterations are true mutations and not NGS artifacts is supported by the following: (i) the point mutations present in ⩾0.2% of NGS reads were confirmed in a reproducibility test; (ii) the same variants were often observed in consecutive samples, (iii) some of the minor-proportion mutations were also noted in individual colonies during FASAY analysis (Supplementary Table 3); this also shows that the mutations are present on separate alleles as FASAY is based on subcloning template molecules; (iv) only one mutation was detected in 56 samples from patients remaining *TP53*-wt throughout disease (40 pretherapy and 16 relapsed samples from Cohort II), and no mutation was observed in any healthy control sample (*n*=20); and finally (v) the molecular profile of additional mutations was similar to that described for the reference cohort^24^ with the common hot spots being the most prevalent mutations.

Despite the similarities between the mutation profile of additional mutations and the reference *TP53*-mutated CLL cohort, we noticed several specificities. The low number of indel mutations among additional mutations can likely be accounted to the NGS methodology itself as it is generally difficult to distinguish minor-proportion 1-nucleotide deletions from background. An interesting observation is the high number of minor-proportion splice-site mutations, predominantly in intron 6. These mutations are often present at the subclonal level; however for a yet unknown reason they only rarely expand to dominant clone. Apart from this, we were not able to find any rule concerning a preferential selection of distinct mutation types. For instance, we recorded patients in which a truncating mutation outgrew the clone carrying hot spot mutation with documented dominant-negative and gain-of-function effect. Therefore, there should be other factors contributing to the preferential selection of particular *TP53*-mutated subclones, for example, mutations in other genes or distinct stimulation by the microenvironment. In addition, an obvious important factor represents deletion 17p, since the wt allele absence may contribute to the selection advantage of a particular subclone. The new 17p deletion accompanying the new *TP53* mutation was found in 8/20 patients and in another two patients a new 17p copy-neutral loss of heterozygosity was noted. However, to determine the exact allele composition of minor subclones carrying different *TP53* mutations would require single-cell analysis, which was beyond the scope of this study.

The surprisingly large number of mutations led us to explore the mechanisms of *TP53* mutagenesis with regard to lymphoid-specific hypermutation machinery. No bias regarding mutations in sequence motifs recognized by activation-induced cytidin deaminase was found. Interestingly, we observed a prevalence of mutations in A:T pairs in patients with mutated *IGHV* compared with unmutated *IGHV*, which was most prominent in A:T>C:G transversions. A similar disproportion was found in a whole-genome sequencing study^30^ and is most likely to be attributed to the operation of error-prone polymerase eta.^31^

The observation that the majority of new dominant mutations are already present before therapy offers the opportunity to predict their expansion later during the disease course and change the patients' care strategy. The obvious prerequisite for such clinical utilization is that persisting minor-proportion *TP53* mutations' existence is not a common phenomenon among patients who do not undergo massive mutation selection. Our long-term observation based on sensitive FASAY analyses in consecutive samples indicates that minor *TP53-*mutated clones may persist in occasional cases without significant expansion. Such a case was also documented here; in one patient we observed only a very slow increase of *TP53*-mutated subclone proportion in consecutive relapses. To explore the general incidence of non-selected mutations, we employed NGS and analyzed 40 pretherapy samples from patients remaining wt after treatment line(s) and observed that non-selected mutations are in fact rare since 39/40 patients were devoid of any mutation.

When considering the applicability of highly sensitive NGS in diagnostics, it is important to bear in mind that (i) the original clone size may be variable and under the detection limit of any method and (ii) the dynamics of the expansion process may vary among individual patients due to competition between CLL subclones and, potentially, also the type of therapy.

As we observed in our study, minor-clone mutations do not have to undergo selection after the first treatment. One may consider that the type of treatment could be critical for clonal selection, with the more intensive regimen being more likely to facilitate clonal selection as we indicated in the previous studies.^32, 33^ However, we document here that in some cases even administrating intensive chemo-immunotherapy resulting in complete remission does not necessarily result in clonal expansion. It is highly likely that there are other factors impacting the selection rate like other genomic defects present either in the *TP53*-mutated subclone itself or in the *TP53*-wt cells.

**Figure 5:**
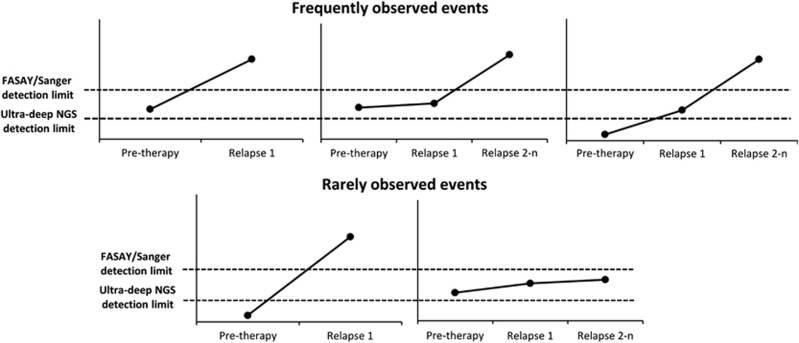
Schematic representation of different scenarios of *TP53*-mutated subclones clonal evolution.

In conclusion, we show in our study that multiple *TP53* clonal evolution scenarios are possible, with some of them being more likely to occur (Figure 5). In cases when a minor-proportion *TP53*-mutated clone(s) is detected, the patient is at high risk of mutation selection by therapy in the first or subsequent relapse, and the presence of the new dominant mutation should be considered as a clearly negative factor impacting the patient's outcome. Moreover, our detailed analysis of *TP53* mutations at the subclonal level at different time points suggests that some patients are intrinsically prone to acquire *TP53* mutations and in the majority of these patients more than one clone carrying a different mutation with a different predisposition for expansion occur. Owing to deep sequencing, it is now technically possible to shift *TP53* mutation identification to time preceding therapy administration. It seems now especially interesting to explore whether similar rules drive the clonal evolution of other recurrently mutated genes in CLL.

## Figures and Tables

**Table 1:** Summary of NGS analysis in patients acquiring a new *TP53* dominant mutation after treatment

*Patient*	*Mutation name*		*FASAY*		*Ultra-deep NGS*—*Sample 1*		*Time from dg to NGS (mo)*	*Time between sample 1 and sample 2 (mo)*	*Therapy before sample 1*	*Therapy between sample 1 and sample 2*	*Cytogenetic aberrations*	*IGHV*	*Disease status*
			*Sample 1*	*Sample 2*	*(%)*	*Total number of mutations*							
*Cohort IA*													
126	c.559+33_54del		neg	pos (40%)	0.52	4	77	23	−	FC	NA→13q-,17p-	unmut	Alive
178	c.488A>G	p.Y163C	neg	pos (18%)	neg	0	45	66	−	FCR/R+D	neg→13q-,17p-	unmut	Alive
199	c.548C>G	p.S183*	neg	pos (24%)	0.57	1	1	81	−	FCR	neg	unmut	Dead
227	c.536A>G	p.H179R	neg	pos (75%)	0.56	1	28	46	−	FC	11q-, 13q-→13q-, 17p-	unmut	Dead
286	c.736A>G	p.M246V	neg	pos (56%)	2.38	1	29	47	−	FCO	13q-	unmut	Alive
503	c.838A>G	p.R280G	neg	pos (13%)	0.20	1	0	31	−	FCR	11q-, 13q-	unmut	Dead
618	c.745A>T	p.R249W	neg	pos (14%)	neg§	1	113	32	−	FCR	11q-, 13q-	unmut	Dead
812	c.817C>T	p.R273C	neg	pos (63%)	0.33	2	0	26	−	FCR	13q-→13q-,17p-	mut	Alive
820	c.743_745del	p.R249del	neg	pos (17%)	2.40	6	0	17	−	FCR	11q-→11q-, cn-LOH 17p	unmut	Dead
837	c.438G>A	p.W146*	neg	pos (17%)	0.85	6	0	24	−	FCR	11q-→11q-, cn-LOH 17p	unmut	Alive
													
*Cohort IB*													
8	c.527G>T	p.C176F	neg	pos (22%)	1.10	4	92	30	Clb, FCR	F/RCHOP	13q-→neg	mut	Dead
149	c.814G>A	p.V272M	neg	pos (50%)	1.11	2	72	24	FCR	FCR	neg	unmut	Dead
161	c.731G>A	p.G244D	neg	pos (18%)	neg	0	14	48	A	FCR	neg→13q-,17p-	unmut	Dead
280	c.818G>A	p.R273H	neg	pos (60%)	1.43	1	15	5	RCHOP	A	neg→13q-,17p-	unmut	Dead
322	c.524G>A	p.R175H	neg	pos (67%)	0.25	1	68	18	Clb	FC	neg→13q-,17p-	unmut	Dead
354	c.844C>T	p.R282W	neg	pos (22%)	0.76	1	77	18	FC	FCR	11q-, 13q-	unmut	Dead
365	c.329G>T	p.R110L	neg	pos (25%)	3.71	4	48	9	FCR/FC	FCR	13q-→13q-,17p-	unmut	Dead
485	c.817C>T	p.R273C	neg	pos (18%)	neg§	2	115	33	Clb	FC	13q-	mut	Alive
542	c.814G>A	p.V272M	neg	pos (35%)	0.76	2	12	22	FCR	FCR	neg→13q-	unmut	Dead
1043	c.844C>G	p.R282G	neg	pos (18%)	0.8	4	32	17	Clb	FCR	13q-	unmut	Alive

Abbreviations: A, alemtuzumab; Clb, chlorambucil; C, cyclophosphamide; F, fludarabine; R, rituximab; FC, fludarabine+cyclophosphamide; FCR, FC+rituximab; FCO, FC+ofatumumab; CHOP, cyclophosphamide; doxorubicin, vincristine, prednisone; RCHOP, CHOP with rituximab; R+D, rituximab+dexamethasone.

Sample 1—sample showing *TP53*-wt status using FASAY; Sample 2—new *TP53* mutation in relapse assessed by FASAY; Cytogenetic aberrations—in case of change cytogenetic aberrations are listed in the format: Sample 1→Sample 2; dg—diagnosis; mo—month; neg—negative; pos—positive; mut—mutated; unmut—unmutated; §—other than the later expanding *TP53* mutation detected (for details, see Supplementary Table 3); cn-LOH 17p—17p copy-neutral loss of heterozygosity involving *TP53* gene detected by Cytoscan Affymetrix arrays.

**Table 2:** Clinical and biological characteristics of patients analyzed using NGS

	*Cohort I*		*Cohort II*		P
	*Dominant TP53 mutation acquisition*		*No TP53 mutated dominant clone after therapy*		
	*Number*	*%*	*Number*	*%*	
Number of patients	20		40		
					
*Rai stage at diagnosis*					
0	6	30	6	15	0.3049
I–II	10	50	23	58	0.5853
III–IV	4	20	11	28	0.7529
					
*Age at diagnosis*					
Median	56.8		59.2		0.3828
Range	45–76		38–77		
					
*Gender*					
Male	15	75	31	78	1.0000
Female	5	25	9	23	
					
*IGHV status*					
Mutated	3	15	4	10	0.6763
Unmutated	17	85	36	92	
					
*I-FISH*^a^ *before treatment*					
Del(17p)	0	0	1	3	1.0000
Del(11q)	6	30	13	34	1.0000
+ 12	0	0	5	13	0.1578
Del(13q)	7	35	13	34	1.0000
Normal	7	35	8	21	0.2199
					
*Follow-up*^b^ *(months)*					
Median	74.8		69.9		0.9808
Range	17–147		15–195		
					
*Number of therapy lines during follow-up*^b^					
Median	2		2		0.4768
Range	1–4		1–5		

Abbreviations: I-FISH, Interphase fluorescent *in situ* hybridization; NGS, next-generation sequencing.

a According to the hierarchical cytogenetics.^20^

b Follow-up: Cohort I—from diagnosis to dominant *TP53* mutation detection; Cohort II—from diagnosis to the last FASAY investigation.

**Table 3:** Consecutive ultra-deep NGS analysis

*Patient*	*FASAY*		*Mutation detected using FASAY*—*major mutation*		*Ultra-deep NGS*						*Time between samples 1 and 2 (mo)*	*Time between samples 2 and 3 (mo)*	*Therapy between samples 1 and 2*	*Therapy between samples 2 and 3*
					*Proportion of major mutation (%)*			*Total number of mutations*						
	*Sample 1*	*Sample 2*			*Sample 1*	*Sample 2*	*Sample 3*	*Sample 1*	*Sample 2*	*Sample 3*				
*Cohort IA*														
178	neg	pos (18%)	c.488A>G	p.Y163C	neg	5.08		0	21		66		FCR/R+D	
199	neg	pos (24%)	c.548C>G	p.S183*	0.57	18.90		1	7		81		FCR	
227	neg	pos (75%)	c.536A>G	p.H179R	0.56	81.50		1	4		46		FC	
286	neg	pos (56%)	c.736A>G	p.M246V	2.38	48.70		1	1		47		FCO	
503	neg	pos (13%)	c.838A>G	p.R280G	0.20	1.46	2.82	1	1	3	31	10	FCR	A
618	neg	pos (14%)	c.745A>T	p.R249W	neg§	7.64		1	4		32		FCR	
812	neg	pos (63%)	c.817C>T	p.R273C	0.33	75.90		2	3		26		FCR	
820	neg	pos (17%)	c.743_745del	p.R249del	2.40	21.50	0.73	6	12	5	17	33	FCR	FCR, RCHOP, VAD
837	neg	pos (17%)	c.438G>A	p.W146*	0.85	64.3		6	7		24		FCR	
														
*Cohort IB*														
8	neg	pos (22%)	c.527G>T	p.C176F	1.10	1.00		4	23		30		F/RCHOP	
354	neg	pos (22%)	c.844C>T	p.R282W	0.76	3.89		1	9		18		FCR	
161	neg	pos (18%)	c.731G>A	p.G244D	neg	11.10		0	3		48		FCR	
485	neg	pos (18%)	c.817C>T	p.R273C	neg§	4.18	5.25	2	20	28	33	18	FC	R+D
542	neg	pos (35%)	c.814G>A	p.V272M	0.76	23.4		2	3		22		FCR	
														
*Cohort II*														
311	neg	neg	c.797G>A	p.G266E	0.55	1.40		1	1		47		FCR, A, R+D	

Abbreviations: A, alemtuzumab; Clb, chlorambucil; C, cyclophosphamide; F, fludarabine; R, rituximab; FC, fludarabine+cyclophosphamide; FCR, FC+rituximab; FCO, FC+ofatumumab; CHOP, cyclophosphamide, doxorubicin, vincristine, prednisone; RCHOP, CHOP+rituximab; R+D, rituximab+dexamethasone; VAD, vincristine, adriamycin, dexamethasone.

Sample 1—sample showing *TP53*-wt status using FASAY; Sample 2—new *TP53* mutation in relapse assessed by FASAY; Sample 3—follow-up sample after mutation detection by FASAY; mo—month; neg—negative; pos—positive; mut—mutated; unmut—unmutated; §—other than the later expanding *TP53* mutation detected (for details, see Supplementary Table 3).
