# Supplementary material for: Detailed analysis of therapy-driven clonal evolution of TP53 mutations in chronic lymphocytic leukemia
Source: Leukemia. 2014 Oct 28;29(4):877–85. doi: 10.1038/leu.2014.297 (PMC4396398; doi:10.1038/leu.2014.297)
Supplement: Supplementary Information [file leu2014297x1.pdf]

Supplementary data

Supplementary methods

Statistical evaluation of detected variants

Up until the alignment step, each sample (represented by paired-end sequencing reads with the length of 2x150 bp) was analyzed using CLC Genomics Workbench version 6.0.4 software (http://www.clcbio.com). Overlapping paired-end reads were merged (with default settings, minimum score 20) and trimmed (with default settings, quality limit 0.05, number of nucleotides between 15 and 500). Read alignment was performed on the unmasked human reference genome (Genome Reference Consortium Human Build 37 patch release 9) with default parameters and similarity fraction of 0.8. Local realignment (CLC Genomics Workbench) was applied before exporting read mappings as bam files, which were further analyzed using R[1] package deepSNV[2,3]. The shearwater algorithm from the deepSNV package was used to compute Bayes classifier based on a betabinomial model for variant calling with prior knowledge[2,3]. Default settings of function "bbb" were applied, and the priors were obtained from the COSMIC v.67 database[4]. As a compound control sample we used the sum of samples, which were negative for *TP53* mutation, as assessed by standard approach (FASAY/Sanger sequencing) (150 samples + 20 healthy control samples). Variants with Bayes factor posterior probability $p \leq 0.01$ were taken into account. To reduce false positives, additional filters were applied: (i) minimum coverage ≥2500 (amplicons that did not reach this number of reads in any nucleotide within coding regions + splice sites were sequenced once again); (ii) number of variant reads ≥10; (iii) mapping quality (QV) ≥30. The genotypes of all patients were then examined for each mutation in the dataset to count the number of times the mutation occurred in the patient cohort. Alternatively, for insertions and ≥2 nucleotide deletions that are undetectable using shearwater algorithm, CLC Genomics Workbench quality based variant calling was applied. The ANNOVAR program was used to annotate variants with gene and exonic function, exon number, position in cDNA and amino acid change (RefSeq) and also for the presence in dbSNP (release 137)[5].

Assessment of reproducibility

To evaluate the reproducibility of NGS, 10 samples were sequenced twice (4 samples with multiple mutations, 3 with one mutation and 3 samples without any mutation). In all cases, the amplicons were prepared in independent PCR reactions and the samples were sequenced in independent runs and pooled with different samples within individual runs. Statistically significant point mutations and insertions/deletions (indels) ≥2 nucleotide were reproducibly detected when present in ≥0.2% of sequencing reads; therefore all such variants present above this threshold were considered in further analyses. In case of 1-nucleotide deletions the detection ability is lower as these may be artificially introduced during the sequencing and alignment process. 1-nucleotide deletions were therefore considered only when present in ≥1% of sequencing reads or when confirmed from consecutive sample. The correctness of this approach was verified by analyzing 20 control samples derived from healthy individuals.

[1] R Core Team (2013). R: A language and environment for statistical computing. R Foundation for Statistical Computing, Vienna, Austria. URL http://www.R-project.org/.

[2] Gerstung M, Papaemmanuil E, Campbell PJ. Subclonal variant calling with multiple samples and prior knowledge. Bioinformatics. 2014. doi:10.1093/bioinformatics/btt750.

[3] Gerstung M, et al. Reliable detection of subclonal single-nucleotide variants in tumour cell populations. Nat. Commun. 2012;3:811.

[4] Forbes SA, et al. COSMIC: mining complete cancer genomes in the catalogue of somatic mutations in cancer. Nucleic Acids Res. 2011;39:D945–D950.

[5] K. Wang, M. Li, and H. Hakonarson, “ANNOVAR: Functional annotation of genetic variants from high-throughput sequencing data,” Nucleic Acids Research, vol. 38, no. 16, pp. e164, 2010.

Supplementary Figures

Supplementary Figure 1. Schematic visualization of sample inclusion criteria

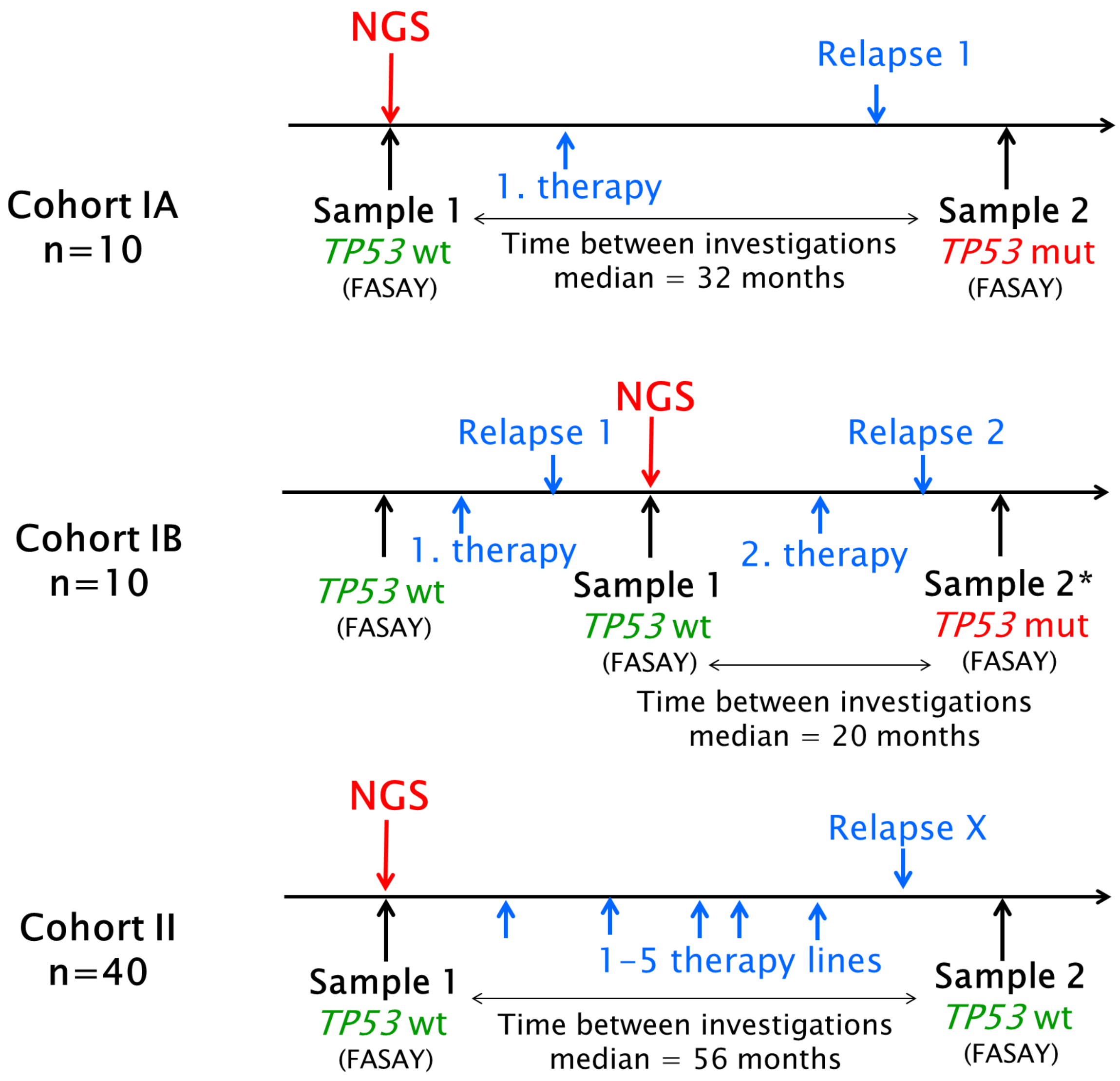

*In one patient (no. 8) *TP53* mutation was detected in the 3rd relapse

Supplementary Figure 2. Distribution of mutations in individual exons and introns

A) All detected mutations

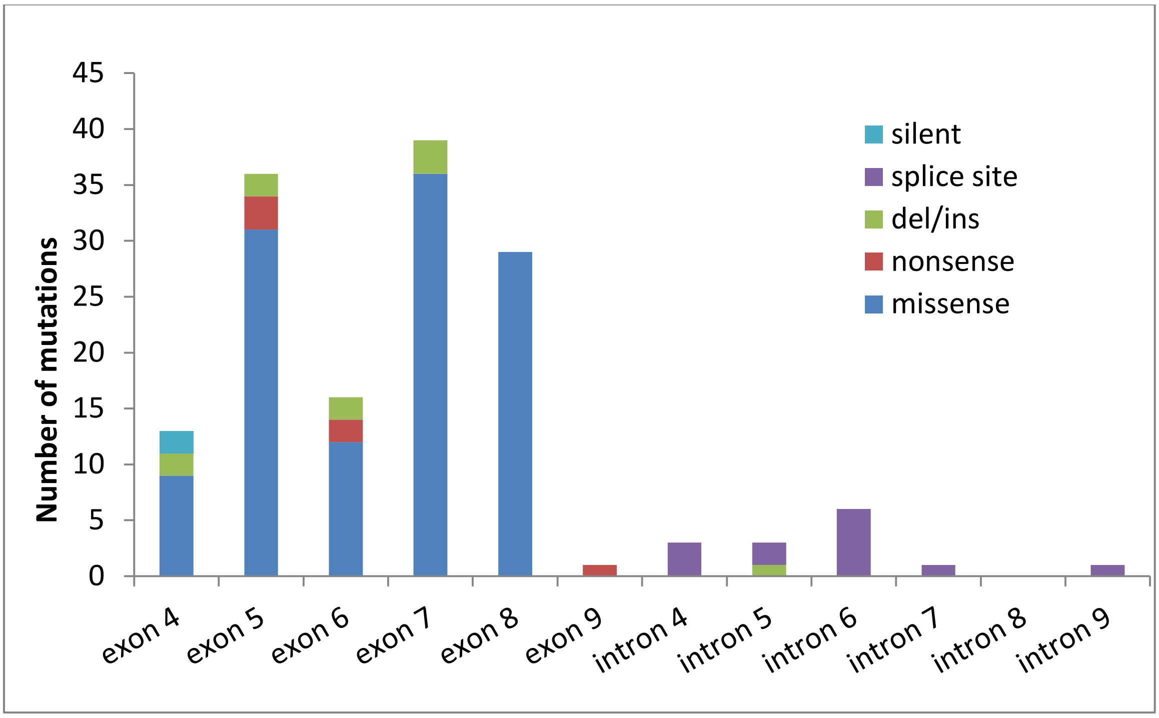

Both major mutations detected by FASAY (n=20) and additional minor-proportion mutations detected by NGS (n=128) are listed.

B) Only minor-proportion mutations detected by NGS (n=128).

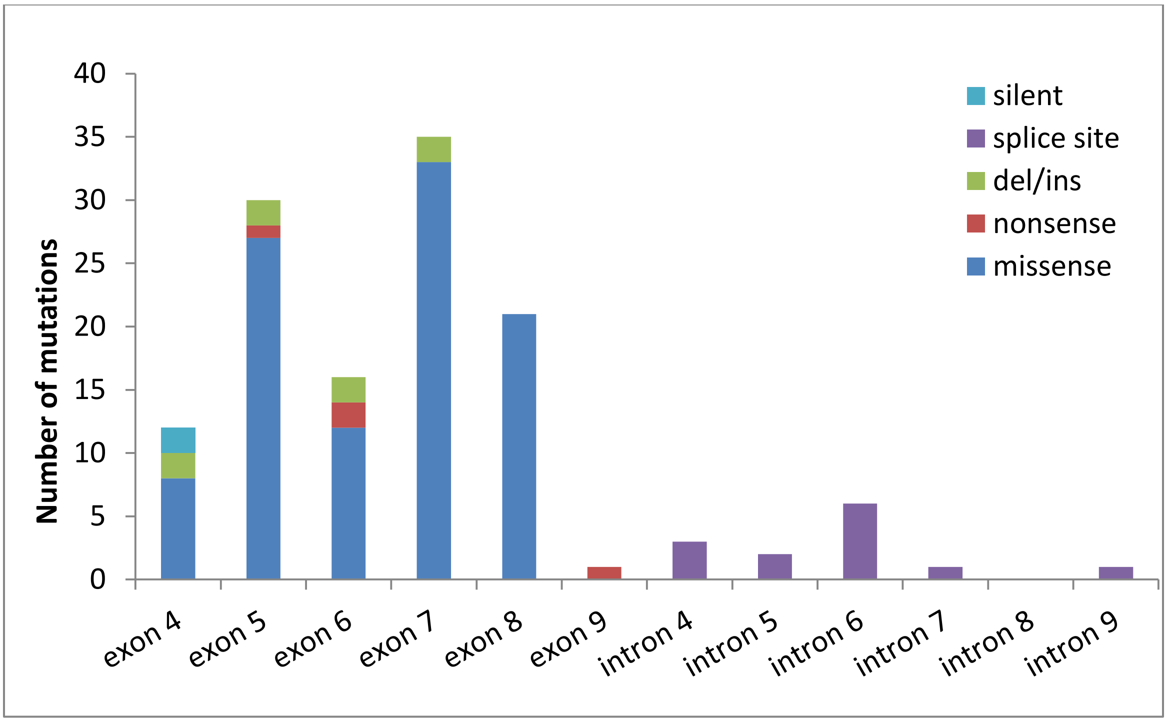

Supplementary Figure 3. Codon distribution of mutations

A) All detected mutations.

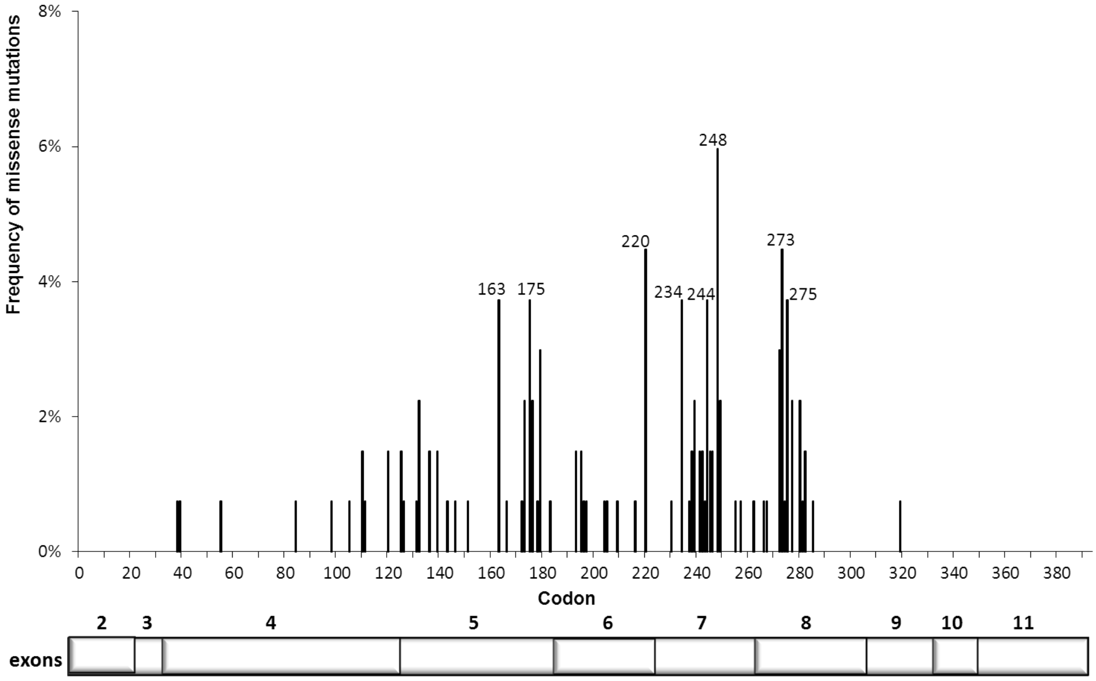

Both major mutations detected by FASAY (n=20) and additional minor-proportion mutations detected by NGS (n=128) are listed. In case of deletions, the first affected codon was considered.

B) Only minor-proportion mutations detected by NGS (n=128).

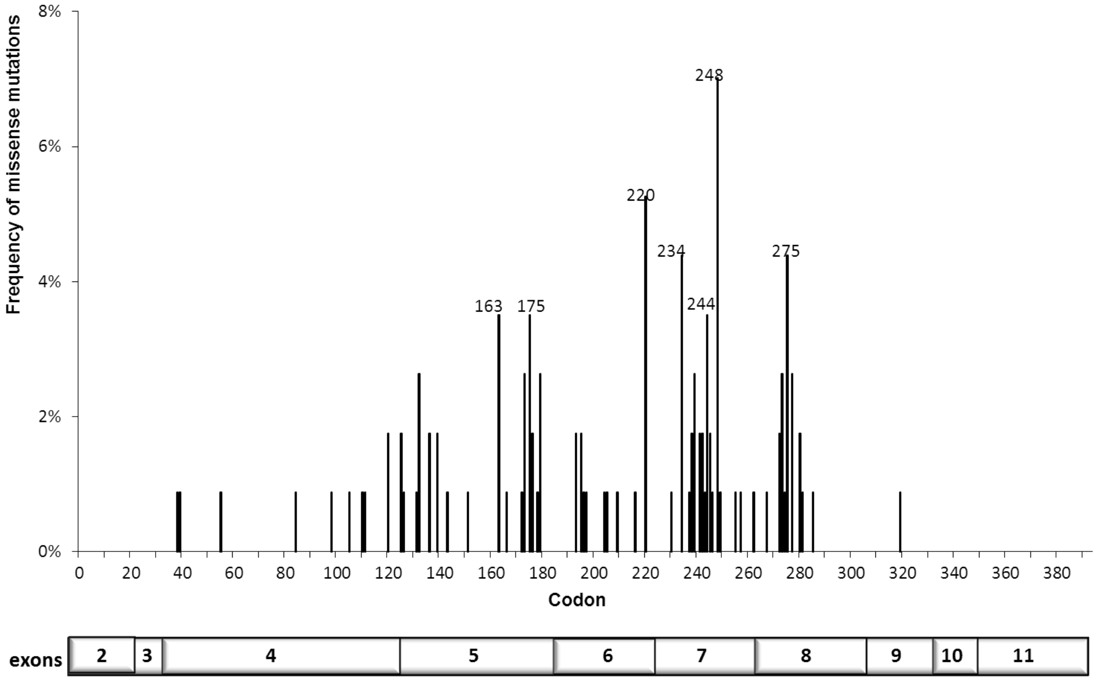

Supplementary Tables

Supplementary Table 1. Custom *TP53* exon-specific primers used for library preparation on MiSeq

Exon	Primer sequence [5'-3']		Amplicon product lenght (bp)
4	F	ACAACGTTCTGGTAAGGACA	511
	R	GACAGGAAGCCAAAGGGTGA	
5-6	F	TGCCGTCTTCCAGTTGCTTT	528
	R	CCCCTACTGCTCACCTGGA	
7	F	CTCCCCTGCTTGCCACA	375
	R	GCCACAGGTTAAGAGGTCCC	
8-9	F	GGGAGTAGATGGAGCCTGGT	487
	R	GCCCCAATTGCAGGTAAAACA	
10	F	TGCATGTTGCTTTTGTACCG	300
	R	GAAGGCAGGATGAGAATGGA	

F – forward
R – reverse

Supplementary Table 2: Characteristics of patients with *TP53*-wt status subjected to consecutive *TP53* mutational analysis

	Untreated		Treated		P
	Number	%	Number	%	
Number of patients	121		209		
RAI stage at diagnosis					
0	69	63%	42	22%	<0.0001
I-II	37	34%	104	55%	0.0003
III-IV	4	4%	42	22%	<0.0001
NA	11		21		
Age at diagnosis					
Median	60		59		0.9981
Range	32-81		33-79		
Gender					
Male	75	62%	155	74%	
Female	46	38%	54	26%	0.0251
IGHV status					
Mutated	71	63%	34	17%	
Unmutated	41	37%	168	83%	<0.0001
NA	9		7		
I-FISH# before treatment					
Del(17p)	1	1%	3	1%	1.0000
Del(11q)	17	14%	61	30%	0.0018
+ 12	11	9%	21	10%	0.8482
Del(13q)	60	50%	54	26%	<0.0001
Normal	31	26%	66	32%	0.2589
NA	1		4		
Treated before first *TP53* examination	0		64	31%	<0.0001
Follow up from diagnosis					
Median	50		61		0.0227
Range	7-166		7-232		
Number of *TP53* examinations	329		744		<0.0001
Number of patients developing *TP53* mutation	1	1%	43	21%	<0.0001

According to the hierarchical cytogenetics (Dohner et al, NEJM. 2000;343(26):1910-6)
NA - not available

Supplementary Table 3. All mutations detected by NGS in individual samplings

						Sample 1			Sample 2			Sample3			
Patient cohort	Patient ID	Mutation name	Mutation name	Mutation type	Gene region	Variant frequency	Variant reads	Total number of reads	Variant frequency	Variant reads	Total number of reads	Variant frequency	Variant reads	Total number of reads	FASAY #
IA	126	c.559+30_51del	p.?	deletion	intron5	0.52%	99	19054	NA			NA			pos
		c.577C>T	p.H193Y	missense	exon6	0.76%	173	22861	NA			NA			
		c.814G>A	p.V272M	missense	exon8	0.78%	163	20814	NA			NA			
		c.824G>T	p.C275F	missense	exon8	0.46%	99	21703	NA			NA			
	178	c.108_112del	p.E38fs	deletion	exon4	neg			0.63%	385	61580	NA			
		c.116C>T	p.A39V	missense	exon4	neg			0.63%	414	65618	NA			
		c.293C>G	p.P98R	missense	exon4	neg			0.25%	176	70241	NA			
		c.375G>A	p.T125T	silent	exon4	neg			0.32%	192	60031	NA			
		c.376-1G>T	p.?	splice	intron4	neg			1.89%	1762	93221	NA			
		c.376T>C	p.Y126H	missense	exon5	neg			0.88%	829	94031	NA			
		c.488A>G	p.Y163C	missense	exon5	neg			5.08%	8480	166883	NA			pos
		c.497C>G	p.S166*	nonsense	exon5	neg			0.23%	398	173990	NA			
		c.524G>A	p.R175H	missense	exon5	neg			5.78%	9425	163112	NA			pos
		c.535C>T	p.H179Y	missense	exon5	neg			1.52%	2695	176842	NA			
		c.559+1G>A	p.?	splice	intron5	neg			0.30%	525	177021	NA			
		c.584T>C	p.I195T	missense	exon6	neg			1.80%	2835	157418	NA			
		c.646G>A	p.V216M	missense	exon6	neg			0.29%	414	144785	NA			
		c.659A>G	p.Y220C	missense	exon6	neg			0.30%	326	107682	NA			
		c.731G>A	p.G244D	missense	exon7	neg			0.22%	186	85617	NA			
		c.731G>T	p.G244V	missense	exon7	neg			0.22%	186	85617	NA			
		c.742C>T	p.R248W	missense	exon7	neg			1.15%	1032	89823	NA			pos
		c.743G>A	p.R248Q	missense	exon7	neg			0.74%	656	88506	NA			
		c.746G>A	p.R249K	missense	exon7	neg			0.49%	415	85580	NA			
		c.764_766del	p.I255fs	deletion	exon7	neg			1.08%	1026	95405	NA			

		c.770T>A	p.L257Q	missense	exon7	neg			0.71%	653	92466	NA			pos
	199	c.164_195del	p.T55fs	deletion	exon4	neg			0.80%	222	27701	NA			
		c.358A>G	p.K120E	missense	exon4	neg			1.04%	232	22318	NA			
		c.548C>G	p.S183*	nonsense	exon5	0.57%	121	21334	18.90%	8100	42870	NA			neg
		c.714T>G	p.C238W	missense	exon7	neg			13.30%	6357	47660	NA			pos
		c.824G>T	p.C275F	missense	exon8	neg			0.23%	68	30186	NA			
		c.830G>T	p.C277F	missense	exon8	neg			0.24%	77	32639	NA			
		c.993+1G>C	p.?	splice	intron9	neg			0.47%	114	24051	NA			
	227	c.536A>G	p.H179R	missense	exon5	0.56%	131	23212	81.50%	29298	35942	NA			pos
		c.536_538del	p.H179_E180delinsQ	deletion	exon5	neg			0.43%	151	35276	NA			
		c.613T>C	p.Y205H	missense	exon6	neg			3.33%	1576	47321	NA			
		c.659A>G	p.Y220C	missense	exon6	neg			8.70%	2526	29048	NA			
	286	c.736A>G	p.M246V	missense	exon7	2.38%	537	22535	48.70%	11905	24467	NA			pos
	503	c.586C>T	p.R196*	nonsense	exon6	neg			neg			0.24%	315	133387	
		c.592_626del	p.E197fs	deletion	exon6	neg			neg			1.95%	2555	131331	
		c.838A>G	p.R280G	missense	exon8	0.22%	66	30295	1.46%	473	32344	2.82%	3825	135848	
	618	c.331C>A	p.L111M	missense	exon4	0.42%	48	11571	1.20%	480	39929	NA			
		c.427G>A	p.V143M	missense	exon5	neg			0.59%	460	78576	NA			
		c.743G>A	p.R248Q	missense	exon7	neg			0.24%	170	71992	NA			
		c.745A>T	p.R249W	missense	exon7	neg			7.64%	5371	70328	NA			pos
	812	c.314G>T	p.G105V	missense	exon4	neg			4.93%	519	10523	NA			
		c.817C>T	p.R273C	missense	exon8	0.33%	82	24601	75.90%	13159	17335	NA			pos
		c.838A>G	p.R280G	missense	exon8	0.20%	61	30504	0.64%	135	21064	NA			
	820	c.375+2T>A	p.?	splice	intron4	1.37%	410	29829	7.18%	1506	20970	15.40%	4339	28241	
		c.524G>A	p.R175H	missense	exon5	3.00%	1118	37287	7.67%	2197	28648	30.70%	12690	41289	pos
		c.578A>T	p.H193L	missense	exon6	neg			0.26%	93	36522	neg			
		c.610G>T	p.E204*	nonsense	exon6	neg			0.26%	80	30250	neg			
		c.722C>A	p.S241Y	missense	exon7	0.27%	99	37391	0.31%	86	27716	neg			
		c.725G>A	p.C242Y	missense	exon7	0.61%	233	38183	1.92%	542	28271	neg			
		c.743G>A	p.R248Q	missense	exon7	neg			0.35%	99	28130	neg			
		c.745_747del	p.R249del	deletion	exon7	2.40%	895	37314	21.50%	6051	28130	0.73%	298	40957	pos

		c.814G>T	p.V272L	missense	exon8	neg			0.24%	94	39008	0.56%	215	38668	
		c.820G>T	p.V274F	missense	exon8	neg			0.37%	140	38401	neg			
		c.824G>A	p.C275Y	missense	exon8	neg			neg			0.36%	141	39653	
		c.853G>A	p.E285K	missense	exon8	0.25%	98	39518	0.90%	402	44667	neg			
	837	c.375G>A	p.T125T	silent	exon4	0.87%	95	10901	3.69%	96	2601	NA			
		c.438G>A	p.W146*	nonsense	exon5	0.86%	113	13086	64.30%	2786	4335	NA			pos
		c.711G>C	p.M237I	missense	exon7	0.40%	134	33637	0.68%	29	4263	NA			pos
		c.713G>A	p.C238Y	missense	exon7	1.56%	516	33130	2.10%	88	4198	NA			
		c.785G>T	p.G262V	missense	exon8	0.29%	74	25893	0.93%	28	3019	NA			pos
		c.817C>G	p.R273G	missense	exon8	neg			1.15%	43	3745	NA			
		c.838A>G	p.R280G	missense	exon8	0.33%	121	37180	1.18%	55	4660	NA			pos
IB	8	c.251C>A	p.A84D	missense	exon4	neg			0.46%	38	8205	NA			
		c.329G>T	p.R110L	missense	exon4	neg			0.40%	44	10914	NA			
		c.359A>T	p.K120M	missense	exon4	neg			0.51%	56	10984	NA			pos
		c.392A>T	p.N131I	missense	exon5	neg			0.51%	146	28448	NA			
		c.395A>C	p.K132T	missense	exon5	neg			0.33%	94	28392	NA			
		c.395A>G	p.K132R	missense	exon5	neg			0.82%	234	28392	NA			pos
		c.406C>G	p.Q136E	missense	exon5	neg			1.16%	340	29406	NA			pos
		c.408A>T	p.Q136H	missense	exon5	neg			0.45%	131	29310	NA			
		c.416A>T	p.K139M	missense	exon5	neg			0.33%	101	31046	NA			
		c.488A>C	p.Y163S	missense	exon5	neg			0.21%	90	42564	NA			
		c.488A>G	p.Y163C	missense	exon5	neg			0.30%	127	42564	NA			
		c.527G>T	p.C176F	missense	exon5	1.09%	322	29615	1.00%	420	42057	NA			pos
		c.560-2A>T	p.?	splice	intron5	0.20%	72	35914	1.23%	527	42834	NA			
		c.583A>T	p.I195F	missense	exon6	neg			0.35%	134	38418	NA			
		c.659A>G	p.Y220C	missense	exon6	neg			3.50%	783	22394	NA			pos
		c.673-2A>C	p.?	splice	intron6	neg			0.54%	112	20807	NA			
		c.673-2A>T	p.?	splice	intron6	neg			0.48%	100	20807	NA			
		c.701A>C	p.Y234S	missense	exon7	neg			0.84%	251	29748	NA			
		c.701A>G	p.Y234C	missense	exon7	0.39%	165	42509	2.27%	674	29748	NA			
		c.716A>G	p.N239S	missense	exon7	neg			0.20%	68	33875	NA			

		c.716A>T	p.N239I	missense	exon7	neg			1.48%	500	33875	NA			pos
		c.722C>T	p.S241F	missense	exon7	neg			0.35%	121	34898	NA			pos
		c.742C>T	p.R248W	missense	exon7	0.26%	121	46590	1.89%	692	36643	NA			
	149	c.733G>A	p.G245S	missense	exon7	0.24%	84	35249	NA			NA			
		c.814G>A	p.V272M	missense	exon8	1.11%	362	32654	NA			NA			pos
	161	c.725G>A	p.C242Y	missense	exon7	neg			0.24%	95	39505	NA			neg
		c.731G>A	p.G244D	missense	exon7	neg			11.10%	4275	38445	NA			pos
		c.800G>A	p.R267Q	missense	exon8	neg			4.33%	1133	26189	NA			neg
	280	c.818G>A	p.R273H	missense	exon8	1.43%	75	5245	NA			NA			pos
	322	c.524G>A	p.R175H	missense	exon5	0.25%	76	30122	NA			NA			pos
	354	c.451C>T	p.P151S	missense	exon5	neg			0.32%	357	110529	NA			
		c.488A>G	p.Y163C	missense	exon5	neg			0.44%	473	107090	NA			
		c.517G>T	p.V173L	missense	exon5	neg			4.31%	4986	115773	NA			pos
		c.523C>G	p.R175G	missense	exon5	neg			3.32%	3539	106685	NA			
		c.659A>G	p.Y220C	missense	exon6	neg			0.56%	446	79559	NA			
		c.742C>T	p.R248W	missense	exon7	neg			0.22%	113	50699	NA			
		c.743G>A	p.R248Q	missense	exon7	neg			0.42%	208	49853	NA			
		c.818G>A	p.R273H	missense	exon8	neg			1.06%	505	47689	NA			
		c.844C>T	p.R282W	missense	exon8	0.76%	185	24253	3.89%	2316	59572	NA			pos
	365	c.329G>T	p.R110L	missense	exon4	3.71%	331	8921	NA			NA			pos
		c.626_627GA	p.R209fs	deletion	exon6	0.77%	52	6794	NA			NA			
		c.729_740del	p.M243fs	deletion	exon7	1.11%	91	8167	NA			NA			pos
		c.842A>G	p.D281G	missense	exon8	1.13%	96	8459	NA			NA			pos
	485	c.376-1G>A	p.?	splice	intron4	neg			0.67%	51	7614	0.73%	336	45749	
		c.395A>G	p.K132R	missense	exon5	neg			0.68%	56	8291	0.40%	213	53037	
		c.416A>C	p.K139T	missense	exon5	neg			0.39%	34	8831	0.40%	235	58187	pos
		c.488A>G	p.Y163C	missense	exon5	neg			0.43%	41	9598	0.27%	209	76807	
		c.517G>T	p.V173L	missense	exon5	neg			0.28%	26	9294	0.37%	298	81489	pos
		c.524G>A	p.R175H	missense	exon5	neg			neg			0.25%	188	76009	
		c.527G>A	p.C176Y	missense	exon5	neg			neg			0.25%	190	75109	
		c.527G>T	p.C176F	missense	exon5	neg			0.34%	29	8443	0.97%	728	75109	

		c.532C>G	p.H178D	missense	exon5	neg			neg			0.21%	165	78114	
		c.535C>T	p.H179Y	missense	exon5	0.54%	97	17904	0.98%	86	8810	0.46%	371	80412	
		c.659A>G	p.Y220C	missense	exon6	neg			0.71%	52	7337	0.70%	332	47663	
		c.673-2A>C	p.?	splice	intron6	neg			0.56%	15	2700	0.84%	202	24088	
		c.673-2A>G	p.?	splice	intron6	neg			neg			0.47%	112	24088	
		c.673-2A>T	p.?	splice	intron6	neg			1.37%	37	2700	3.77%	908	24088	
		c.700T>C	p.Y234H	missense	exon7	neg			neg			0.29%	96	33723	
		c.701A>C	p.Y234S	missense	exon7	neg			neg			0.63%	213	33796	
		c.701A>G	p.Y234C	missense	exon7	neg			1.83%	66	3610	4.14%	1399	33796	pos
		c.716A>G	p.N239S	missense	exon7	neg			0.28%	11	3945	neg			
		c.730G>A	p.G244S	missense	exon7	neg			neg			0.23%	91	39365	
		c.730G>T	p.G244C	missense	exon7	neg			0.51%	20	3950	2.27%	894	39365	
		c.733G>C	p.G245R	missense	exon7	neg			0.26%	10	3927	0.86%	342	39801	pos
		c.736A>G	p.M246V	missense	exon7	neg			neg			0.25%	99	40158	
		c.743G>A	p.R248Q	missense	exon7	neg			0.45%	18	4040	0.21%	84	40452	
		c.783-1G>A	p.?	splice	intron7	neg			neg			0.29%	105	35820	
		c.817C>T	p.R273C	missense	exon8	neg			4.18%	211	5048	5.25%	2506	47693	pos
		c.818G>A	p.R273H	missense	exon8	0.25%	44	17964	0.57%	29	5083	0.35%	165	47526	
		c.823T>C	p.C275R	missense	exon8	neg			0.62%	33	5332	1.03%	529	51168	pos
		c.824G>T	p.C275F	missense	exon8	neg			0.32%	17	5343	neg			
		c.830G>A	p.C277Y	missense	exon8	neg			0.44%	25	5742	neg			pos
		c.830G>T	p.C277F	missense	exon8	neg			neg			0.32%	175	55005	
		c.955A>T	p.K319*	nonsense	exon9	neg			neg			0.21%	108	50848	
	542	c.516delT	p.V173*	deletion	exon5	0.63%	76	12000	3.31%	735	22204	NA			
		c.672+1G>A	p.?	splice	intron6	neg			0.27%	39	14512	NA			
		c.814G>A	p.V272M	missense	exon8	0.76%	328	43329	23.40%	6553	27964	NA			pos
	1043	c.515T>A	p.V172D	missense	exon5	0.30%	74	24329	NA			NA			
		c.659A>G	p.Y220C	missense	exon6	0.87%	154	17783	NA			NA			
		c.688A>G	p.T230A	missense	exon7	0.68%	148	21761	NA			NA			
		c.844C>G	p.R282G	missense	exon8	0.83%	71	8540	NA			NA			pos
II	311	c.797G>A	p.G266E	missense	exon8	1.40%	889	63700	1.40%	889	63700	NA			

# - FASAY - mutation detected in at least one red FASAY colony in at least one sampling
pos – positive, neg – negative, NA - not available
